# Effects of Niacin on Resistance to Enterotoxigenic *Escherichia coli* Infection in Weaned Piglets

**DOI:** 10.3389/fnut.2022.865311

**Published:** 2022-04-29

**Authors:** Rui Zhen, Junsen Feng, Dongsheng He, Yibo Chen, Tianbao Chen, Weiyou Cai, Yunxia Xiong, Yueqin Qiu, Zongyong Jiang, Li Wang, Hongbo Yi

**Affiliations:** ^1^State Key Laboratory of Livestock and Poultry Breeding, Ministry of Agriculture Key Laboratory of Animal Nutrition and Feed Science in South China, Guangdong Public Laboratory of Animal Breeding and Nutrition, Guangdong Key Laboratory of Animal Breeding and Nutrition, Institute of Animal Science, Guangdong Academy of Agricultural Sciences, Guangzhou, China; ^2^College of Veterinary Medicine, South China Agricultural University, Guangzhou, China

**Keywords:** nicotinic acid, weaned piglets, ETEC, antibacterial peptides, HDACs

## Abstract

Nicotinic acid (NA) has been used to treat different inflammatory disease with positive influence, the mechanisms by which NA exerts its anti-inflammatory effects remain largely undefined. Here we proposed a new hypothesis that NA manipulated endogenous antimicrobial peptides (AMPs) which contributed to the elimination of enterotoxigenic *Escherichia coli* (ETEC) K88, and thus affects the alleviation of inflammation. Therefore, an experiment in weaned piglets treated with 40 mg NA for 3 days before ETEC K88 challenge was designed to investigate the effects of NA on resistance to enterotoxigenic *E. coli* infection in weaned piglets. Twenty-four weaned piglets were randomly assigned to 1 of 4 treatments based on weight and sex. The control and NA treated groups were administered 20 mL normal saline or 20 mL NA solution. The K88 challenged and NA treated plus K88 challenged groups were administered 20 mL normal saline or 20 mL nicotinic acid solution once daily for 3 consecutive days. On the fourth day, the K88 and K88 + NA groups were treated with oral administration of 4 × 10^9^ cfu/mL ETEC K88. The results showed that NA alleviated the clinical symptoms of weaned piglets infected with ETEC K88. NA significantly reduced the amount of ETEC K88 in the spleen and liver (*P* < 0.05). The intestinal morphological damage caused by ETEC K88 infection was alleviated by NA in weaned piglets. In addition, NA significantly alleviated the expression of inflammatory cytokine [Interleukin-6 (IL-6), Interleukin-8 (IL-8), tumor necrosis factor-α (TNF-α)] in the serum and intestines of weaned piglets infected with ETEC K88 (*P* < 0.05). NA significantly increased the content of secretory IgA (SIgA) and the expression of antimicrobial peptides [porcine β defensin-2 (pBD2), protegrin1-5 (PG1-5) and PR39] in intestines of weaned pigs. NA increased the diversity of microflora in colonic contents, while NA significantly reduced the relative abundance of *Bacteroidetes, Bacteroidales*, and *Bacteroidia* in weaned piglets infected with ETEC K88 (*P* < 0.05). Furthermore, the NA group significantly reduced the level of HDAC7 in jejunum (*P* < 0.05) and increased the level of SIRT1 in the colon compared with the Control group. Moreover, NA significantly increased the levels phosphorylation of histone H3 at Ser10 (pH3S10) in ileum and the levels of acetylation of lysine 9 on histone 3 (acH3K9) and acH3K27 in colon (*P* < 0.05) in weaned piglets infected with ETEC K88 (*P* < 0.05). In conclusion, NA can alleviate the clinical symptoms, the damage of intestinal morphology, and intestinal inflammation in weaned piglets infected ETEC K88 through enhancing the expression of endogenous AMPs by associating the histone acetylation modification.

## Graphical Abstract

Nicotinic acid could improve intestinal antimicrobial peptides to enhance resistance of weaned piglets to *E. coli* infection by regulating intestinal microflora and its metabolites, histone deacetylase SIRT1 and HDAC7, modification sites such as acH3K9, acH3K27, and pH3S10 in the promoter region.

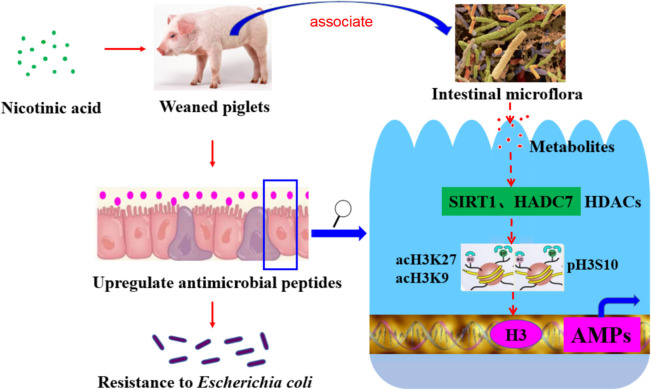

## Introduction

Infants and other mammalian neonates often suffer from diarrhea during weaning, which is the leading killer of children under 5 years of age in developing countries all over the world (1, 2). Studies have shown that the piglets are usually faced with some problems such as physical or mental disorders, changes in small intestinal structure, disturbed intestinal microbiota and diminished immune responses during weaning (3, 4), which will easily lead to diarrheal disease caused by the invasion of various pathogenic bacteria, especially enterotoxigenic *E. coli* (ETEC). ETEC post-weaning diarrhea, also named as postweaning enteric colibacillosis, is a crucial factor causing mortality of nursery pigs in the global swine production. The infection of ETEC in nursery pigs may induce diarrhea during the first 1 or 2 weeks of postweaning periods usually resulting in dehydration, reduced weight gain, and death (5). Therefore, it is extremely urgent to find an effective way to improve the disease resistance of weaned piglets.

Nicotinic acid (NA), also known as Vitamin B3, is one of the most important water-soluble B vitamins in mammals, and widely used as a feed additive in modern animal husbandry. Previous studies had shown that NA played an important role in anti-pellagra and regulation of cellular energy metabolism (6). As reported, nicotinamide treatment could ameliorate the course of bacterial and chemical induced colitis by enhancing neutrophil-specific antibacterial clearance (7). What’s more, accumulating evidence from mouse suggested that NA alleviated intestinal mucosal inflammation and enhanced the expression of endogenous antimicrobial peptides in intestinal epithelium (8). Endogenous antimicrobial peptides are an important part of innate immunity in animals. More and more evidence shows that antimicrobial peptides play a key role in pathogen resistance and immune regulation (9, 10). However, there are few studies on the mechanism of NA regulating intestinal antimicrobial peptides to enhance resistance ETEC infection in weaned piglets.

Thus, a model of ETEC K88 infected early weaned piglets was established, aiming to investigate the mechanism of NA regulating intestinal immunity to enhance resistance of weaned mammalian neonates, as assessed by analyzing intestinal morphology, intestinal immune responses, microbial community and metabolites, and the histone acetylation modification in this study.

## Materials and Methods

### Animals, Experimental Design, and Sample Collection

The animal protocol was approved by the Animal Care Committee of the Institute of Animal Science, Guangdong Academy of Agricultural Sciences. Twenty-four weaned piglets (Duroc × Landrace × Yorkshire, age of 21 d) were randomly assigned to 1 of 4 treatments based on BW and sex, each treatment with 6 piglets and 1 piglet per pen in a temperature-controlled room. The control (Control) and NA-treated (NA) groups were administered 20 mL normal saline or 20 mL NA solution (40 mg NA was dissolved in equal volume of normal saline). The K88 challenged (K88) and NA-treated plus K88 challenged (K88 + NA) groups were administered 20 mL normal saline or 20 mL nicotinic acid solution once daily for 3 consecutive days. On the fourth day, the K88 and K88 + NA groups were treated with oral administration of 4 × 10^9^ cfu/mL ETEC K88. All piglets were provided with access to water *ad libitum*. The piglets were checked daily for signs of diarrhea. At the end of experiment, the animals were individually weighed, weight loss of piglets was counted. Samples of the duodenum, jejunum, ileum, and colon were collected for analysis. Serum was obtained from the separation gel coagulation promoting tubes after centrifugation at 3 000 × *g* for 15 min at 4°C and stored immediately at −20°C.

### Bacterial Plate Counting Analysis

Pathogenic *E. coli* K88 was preserved by the laboratory of Institute of Animal Sciences, Guangdong Academy of Agricultural Sciences. About 2.5 g liver and spleen tissues of pigs in 2.25 mL sterilized buffer liquid, and then homogenized. Aliquots of 10 mL of the dilutions to be analyzed are placed into LB agar medium flat plate, test three parallel plates for each sample. Plates are inverted and incubated for 18–24 h at 37°C in a constant temperature incubator. Calculate the CFUs of bacterial transfer as the weighted mean from the successive dilutions, which contain between 30 and 300 colonies. The calculate result is the weighted means of the successive dilution multiply by dilution factor.

### Intestinal Morphology Analysis

Formalin-fixed duodenum, jejunum, ileum, and colon samples were embedded in paraffin wax. Segment cross sections were microtomed at approximately 5 μm thick and stained with hematoxylin and eosin (H&E). In each section, villus height and associated crypt depth were measured using a DM3000 microscope (Leica Microsystems, Wetzlar, Germany). Images were obtained *via* using a DM3000 microscope (Leica Microsystems, Wetzlar, Germany). For each section, measurements of 6, well-orientated and intact villi were examined in each piglets’ duodenum, jejunum, and ileum. In the end, the mean villus height was then calculated per piglet with Image-Pro software (Media Cybernetics, Rockville, MD, United States). Histopathologic damage scores were determined according to the statement in Feng’s publication (11).

### Immunoglobulins, Cytokines, and Biochemistry Measurements

The concentrations of secretory IgA (SIgA) in the jejunal and ileal mucosa of piglets were determined using the commercially available enzyme-linked immunosorbent assay (ELISA) kits from TSZ ELISA (Framingham, MA) according to the manufacturer instructions. The concentrations of IgM, IgA, IgG, IL-6, IL-8, TNF-α, and IFN-β in serum of piglets were determined using ELISA kits from Nuoyuan Co., Ltd. (Shanghai, China). The concentrations of SIgA, IgM, IgA, IgG, IL-6, IL-8, TNF-α, and IFN-β were quantified by using a BioTek Synergy HT microplate reader (BioTek Instruments, Winooski, VT), and absorbance was measured at 450 nm. Serum samples were analyzed by ABX Pentra 400 Clinical Chemistry Analyzer (Horiba ABX, Northampton, United Kingdom) for total protein (TP), albumin (ALB), globulin (GLOB), glucose (GLU), lactic dehydrogenase (LDH), blood urea nitrogen (BUN), aspartate aminotransferase (AST), and alkaline phosphatase (ALP).

### Analysis of Intestinal Microbiota *via* 16S rRNA Gene Sequencing

The contents in the colon of the piglets were aseptically collected, and the total DNA of the colonic contents was extracted using a DNA Kit (SimGEN, Hangzhou, China) according to the instructions provided by the manufacturer. Subsequently, the purity and yield of the DNA samples were quantified using a NanoDrop 1000 (Thermo Fisher Scientific, Waltham, MA, United States) spectrophotometer. Then, twenty-four samples (*n* = 6) were sequenced on an Illumina HiSeq PE250 platform provided by Novogene (Beijing, China). Paired-end reads from the original DNA fragments were merged by using FLASH. Clustering was performed using the UPARSE pipeline, and sequences were classified into different operational taxonomic units (OTUs) based on the sequence similarity cut-off value (i.e., 97%). Lastly, the diversity and composition of the bacterial communities were determined by α and β diversity according to Novogene’s recommendations. At the phylum, class and order levels, LEfSe was used to identify metagenomic biomarkers, while linear discriminant analysis (LDA) was used to estimate the effect of abundance of each species on the difference between groups.

### Untargeted Metabolomic Analysis of Colonic Contents

Metabolite extractions: equal volume of liquid samples was dried on a freeze-drier, then 0.5 mL cold extraction solvent methanol/acetonitrile/H_2_O (2:2:1, v/v/v) was added to the sample, and adequately vortexed. After vortexing, the samples were incubated on ice for 20 min, and then centrifuged at 14,000 *g* for 20 min at 4°C. The supernatant was dried in a vacuum centrifuge. For LC-MS analysis, the samples were re-dissolved in 100 μL acetonitrile/water (1:1, v/v) solvent and transferred to LC vials. LC-MS analysis, data analysis and bioinformatics analysis were performed according to the method in our previous study (12).

### Relative Quantitative in Real-Time PCR

Total RNA was extracted from the intestinal tissue samples using Trizol reagent (Invitrogen, Carlsbad, CA, United States). The amount of RNA extracted was determined and its purity was verified using NanoDrop 1000 (Thermo Fisher Scientific, Waltham, MA, United States). Contaminant DNA was removed by gDNA Eraser (Takara, Dalian, China). The cDNA was generated using 1 μg aliquot of total RNA with a PrimeScript RT Reagent Kit (Takara). Synthesized cDNA was stored at –20°C prior to real-time PCR analysis.

Real-time PCR was performed using a CFX Connect Detection system (Bio-Rad, Hercules, CA, United States). The sequences of primers used in this study were listed in Table 1. Primers for specific porcine genes were synthesized by Biotechnology Inc. The cDNA was amplified with SYBR^®^ Premix DimerEraser™ (Takara Biotechnology Inc., Kusatsu, Japan) containing 4-μL 20-fold diluted cDNA, 0.5 μL primers F (10 μM), 0.5 μL primers R (10 μM), 5 μL iTaq Universal SYBR Green Supermix (Bio-Rad), The PCR amplification was performed using the following conditions: 95°C for 30 s, followed by 40 cycles at 95°C for 5 s, 60°C for 30 s, and 72°C for 30 s. The melting curves were systematically analyzed to evaluate the specificity after each run. All reactions were conducted in triplicate. To evaluate the relative quantification of mRNA expression, the cycle threshold (CT) values of the target genes were normalized to the CT-values of the β-actin, and the results were presented as fold changes using the 2^–ΔΔ^ ^Ct^ method.

**TABLE 1:** Primers for the real-time PCR analysis.

Gene	Sequence (5′-3′)^1^
IL-6	F: TGGCTACTGCCTTCCCTACC R: CAGAGATTTTGCCGAGGATG
IL-8	F: TTCGATGCCAGTGCATAAATA R: CTGTACAACCTTCTGCACCCA
pBD-2	F: CCAGAGGTCCGACCACTACA R: GGTCCCTTCAATCCTGTTGAA
TNF-α	F: CCAATGGCAGAGTGGGTATG R: TGAAGAGGACCTGGGAGTAG
IFN-β	F: AGTGCATCCTCCAAATCGCT R: GCTCATGGAAAGAGCTGTGGT
pG1-5	F: GTAGGTTCTGCGTCTGTGTCG R: CAAATCCTTCACCGTCTACCA
PR-39	F: CTTCCCAGTAGAGGCATGTTATT R: GCCACAGTTTGAGGTGATTTG
β-actin	F: CTGCGGCATCCACGAAACT R: AGGGCCGTGATCTCCTTCTG

*^1^F = forward, R = reverse.*

### Western Blot Analysis

Total protein was extracted from intestinal tissue samples using lysis buffer (KeyGEN, Nanjing, China). The protein concentrations of each sample were calculated with the BCA protein assay kit. Protein was used for western blot analysis, after adding 6× concentrated sample buffer (0.5M Tris, 30% glycerol, 10% SDS, 0.6M DTT, 0.012% bromophenol blue) and heating the samples for 5 min at 95°C. Proteins in supernatants were separated by sodium dodecyl sulfate–polyacrylamide gel electrophoresis (SDS-PAGE) and then transferred to polyvinylidene fluoride membranes (PVDF) membranes. Next, the membranes were incubated with the primary antibodies for β-actin (Abcam, MA, United States), SIRT1, HDAC7, acH3, acH3K9, acH3K27, and pH3S10 (Abcam, MA, United States) overnight at 4°C after blocked with Tris-buffered saline containing 0.1% Tween-20 (TBS-T) and 5% low-fat milk blocked for 1 h at RT. After washing for 1 h in TBS-T five times, the membranes were incubated for 1 h at RT with horseradish peroxidase (HRP) secondary antibodies. Protein immunoreactive bands were photographed. Each special banding gray value was digitized using ImageJ software, and the gray value of target protein was divided by internal reference β-actin.

### Statistical Analysis

Statistical significance analysis of the experimental data was determined by the analysis of variance (ANOVA) with Duncan’s multiple range test using SPSS 18.0 software (SPSS Inc., Chicago, IL, United States). Values are given as means ± SEM. The difference was considered to be significant at *P* < 0.05.

## Results

### Effects of Nicotinic Acid on Body Weight Loss, Clinical Symptoms, and Intestinal Morphology Weaned Piglets Infected by Enterotoxigenic *Escherichia coli*

**FIGURE 1:**
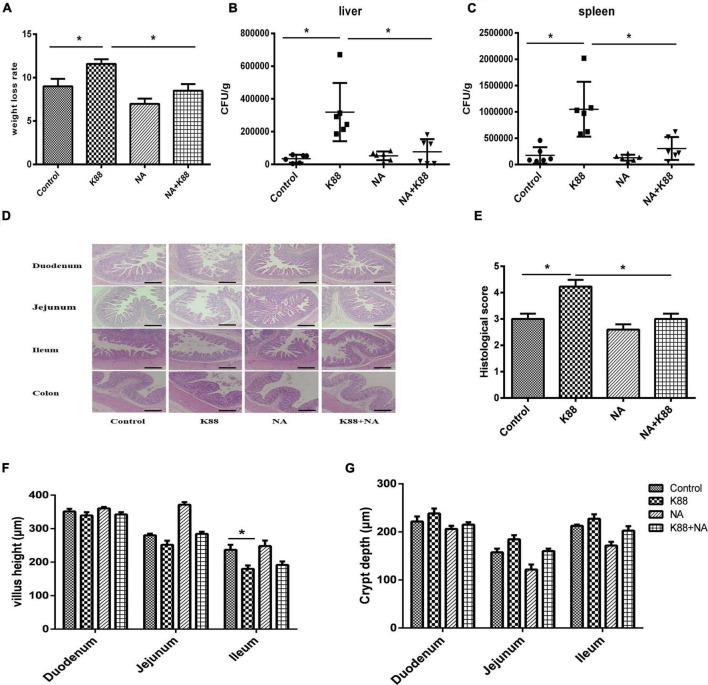
Effects of niacin on body weight loss rate and intestinal morphology caused by *Escherichia coli* infection of weaned piglets. The weight loss rate of piglets was showed **(A)**, the number of CFU of the bacteria transfer to the liver **(B)** and spleen **(C)** after infection tissues by bacterial plate counting analysis. Stained with H&E (bars, 500 μm) **(D)**. Histological scores were determined as described in the Materials and Methods **(E)**. Villous height in the jejunum, duodenum, and ileum **(F)**. Crypt depth in the jejunum, duodenum, and ileum **(G)**. All data are expressed as the mean ± SEM. *P < 0.05.

We found that NA effectively attenuated the rate of weight loss (Figure 1A) and diarrhea in weaned piglets. To investigate whether the positive effects of nicotinic acid treatment on clinical symptoms were connected to a reduction of the *E. coli* load in the gut, bacterial plate counting analysis of colony-forming units (CFUs) in the liver and spleen tissues was performed. Compared with the Control group, the CFU counts of *E. coli* in the liver and spleen of piglets was significantly increased in the K88 group (*P* < 0.05). The K88 + NA group significantly decreased the CFU counts of bacteria in liver and spleen tissues compared with the K88 group (*P* < 0.05) (Figures 1B,C). Then, we investigated the effects of nicotinic acid on the intestinal morphology of piglets, the results showed that nicotinic acid not only improved intestinal morphology, but also improved intestinal integrity compared with the Control group (*P* < 0.05) (Figures 1D–G).

### Effects of Nicotinic Acid on the Inflammation in Intestinal Tissues and Serum of Weaned Piglets Infected by Enterotoxigenic *Escherichia coli*

**FIGURE 2:**
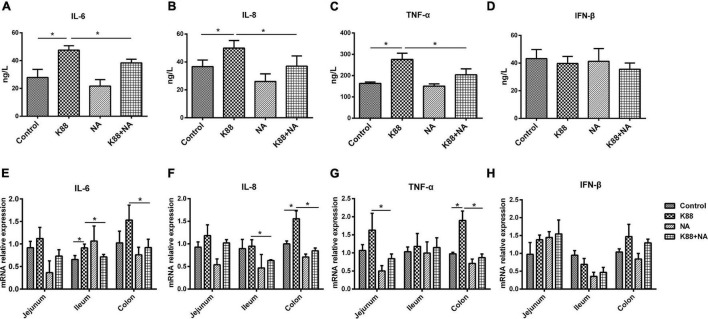
Nicotinic acid ameliorated the inflammation caused by *E. coli* infection in intestinal tissues and serum of weaned piglets. The level of serum IL-6 **(A)**, IL-8 **(B)**, TNF-α **(C)**, and IFN-β **(D)** were determined *via* ELISA. The relative mRNA expression levels of IL-6 **(E)**, IL-8 **(F)**, TNF-α **(G)**, and IFN-β **(H)** in the jejunum, ileum, and colon were determined *via* real-time PCR. All data are expressed as the mean ± SEM. *P < 0.05.

To determine the anti-inflammatory effect of NA on the resistance of weaned piglets to ETEC K88 infection, the expression and secretion of the inflammatory cytokines (IL-6, IL-8, TNF-α, and IFN-β) were evaluated in intestinal tissues and serum. Compared with the Control group, there were higher levels of serum IL-6, IL-8, and TNF-α in the K88 group (*p* < 0.05). However, the levels of serum IL-6, IL-8, and TNF-α were significantly lower in K88 + NA group compared with the K88 group (*p* < 0.05) (Figures 2A–C). Compared with the Control group, the K88 group significantly increased the expression of ileal inflammatory cytokines IL-6 and colonic inflammatory cytokines IL-8 and TNF-α (*p* < 0.05). However, the expression of inflammatory cytokines IL-6 and IL-8 in the ileum and colon and the expression of TNF-α in jejunum and colon were significantly lower in the K88 + NA group compared with the K88 group (*p* < 0.05) (Figures 2E–G). There were no significant differences in the change of IFN-β in intestinal tissues and serum (*p* > 0.05)(Figures 2D,H). Taken together, these data indicated that with NA treatment ameliorated the inflammation caused by ETEC K88 infection.

### Effects of Nicotinic Acid on Serum Biochemistry and Immunoglobulins in Weaned Piglets Infected by Enterotoxigenic *Escherichia coli*

**FIGURE 3:**
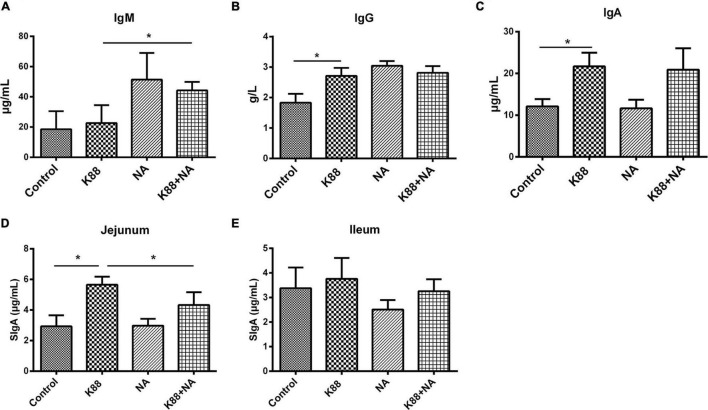
Effects of nicotinic acid on serum levels of immunoglobulins caused by *E. coli* infection of weaned piglets. The serum levels of immunoglobulins IgM **(A)**, IgG **(B)**, IgA **(C)**, and SIgA in jejunum **(D)** and ileum **(E)** mucosa were determined by ELISA. All data are expressed as the mean ± SEM. *P < 0.05.

The effects of NA on serum biochemistry and immunoglobulins caused by *E. coli* infection of weaned piglets were, respectively shown in Figure 3 and Table 2. Compared with the K88 group, the level of serum lactate dehydrogenase (LDH) in the K88 + NA group was significantly increased (*p* < 0.05). The K88 group significantly reduced blood glucose (GLU) content in serum piglets compared with the normal control group (*p* < 0.05). However, the K88 + NA group can significantly improve serum glucose (GLU) content compared with the K88 group of piglets (*p* < 0.05). In the aspect of immunoglobulins (Figures 3A–C), the K88 group had no significant effect on serum IgM, but significantly increased serum IgG and IgA compared with the Control group (*P* < 0.05). Compared with K88 group, the serum IgM in the K88 + NA group was significantly increased (*P* < 0.05). The content of SIgA in jejunum and ileum mucosa of piglets was measured by ELISA (Figures 3D,E). The results showed that the K88 group significantly increased the content of SIgA in jejunum mucosa of piglets compared with the Control group. Compared with the K88 group, the K88 + NA group significantly ameliorated the increase of SIgA in jejunum mucosa of piglets (*P* < 0.05).

**TABLE 2:** Effect of nicotinic acid treatment on serum biochemistry parameters caused by *Escherichia coli* infection of weaned piglets.

Item	Control	K88	NA	K88 + NA	SEM	*p*-value
TP(g/L)	48.60^b^	54.47^a^	53.05^ab^	53.15^ab^	0.96	0.605
ALB(g/L)	37.10	40.03	41.45	38.05	0.75	0.058
GLOB(g/L)	11.50^b^	14.43^a^	11.60^b^	15.10^a^	0.56	0.623
A/G	3.47^ab^	2.83^ab^	3.63^a^	2.58^b^	0.17	0.086
AST(U/L)	44.50	38.83	43.83	40.83	2.87	0.553
ALP(U/L)	357.80^ab^	317.17^bc^	397.83^a^	264.17^c^	14.13	0.170
LDH(U/L)	531.20^ab^	457.83^b^	541.83^ab^	598.83^a^	22.75	0.307
BUN(mmol/L)	3.66^b^	4.58^ab^	4.02^b^	5.63^a^	0.25	0.097
GLU(mmol/L)	5.71^a^	4.32^b^	5.66^a^	5.75^a^	0.16	0.789

*^a,b,c^ Means within a row with different superscript letter differ (p < 0.05).*

### Effects of Nicotinic Acid on the Microbial Community in Colonic Contents of Weaned Piglets Infected by Enterotoxigenic *Escherichia coli*

**FIGURE 4:**
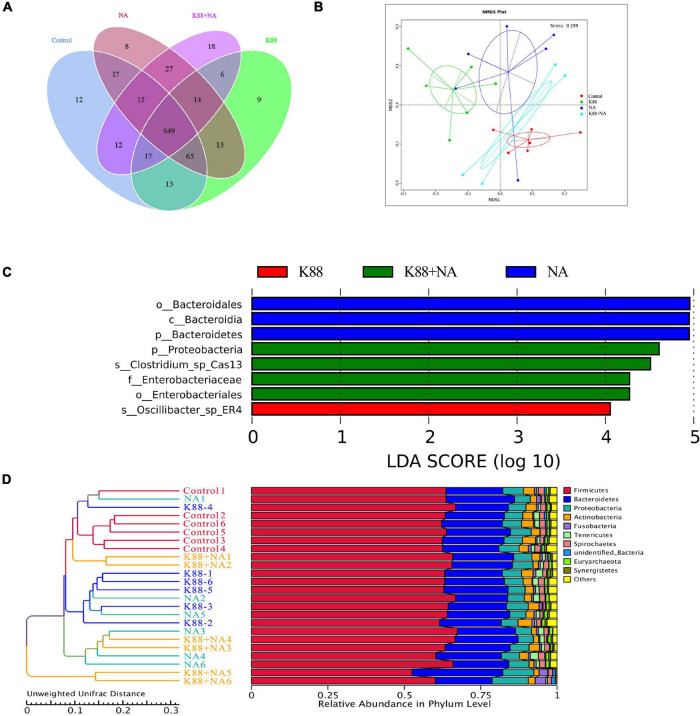
Nicotinic acid improved the bacterial community caused by *E. coli* infection in colonic contents of weaned piglets. The bacterial communities in the colonic contents of weaned piglets were investigated using Illumina sequencing of the 16S rRNA gene. Venn diagram shows the common and special OTUs distribution among the four groups **(A)**, non-metric multidimensional scaling (NMDS) based on operational taxonomic unit levels **(B)**, the LDA score analysis (LDA score ≥ 4) in four groups **(C)**, UPGMA Clustering was conducted based on Unweighted Unifrac distance **(D)**.

**FIGURE 5:**
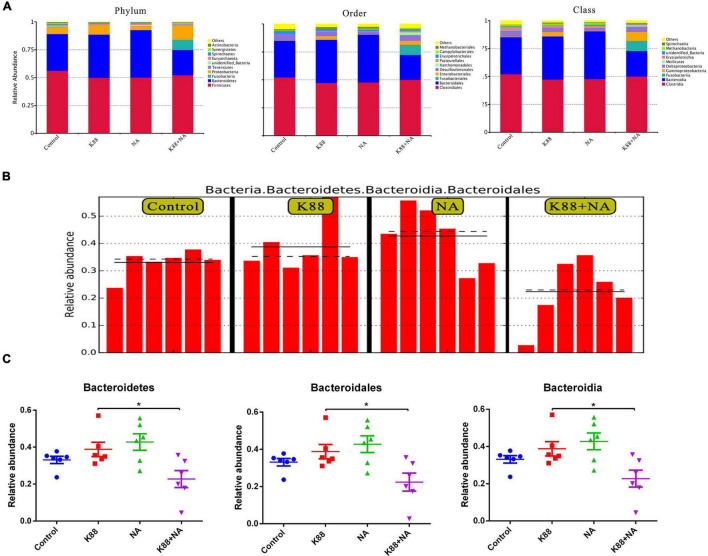
Nicotinic acid improved the classification of the bacterial community composition caused by *E. coli* infection in colonic contents of weaned piglets. The relative abundance of top 10 in phylum, order and class **(A)**, the LEfSe analysis identified the biomarker bacterial species **(B)**, the significantly different species at each level **(C)**. *P < 0.05.

We evaluated the effects of NA on the composition of microbiota caused by *E. coli* infection in the colonic contents of weaned piglets using Illumina sequencing of the 16S rRNA (Figures 4, 5). The common and special OTUs distribution among the four groups was presented by Venn diagram (Figure 4A). As shown in NMDS plot (Figure 4B), the K88 + NA group formed a distinct cluster clearly separated from the K88 group. *Bacteroidetes* and Firmicutes were the two most abundance bacterial phyla in all samples (Figure 4C). By LDA score analysis, we identified a total of 8 discriminative species among the four groups (Figure 4D). Only oscillibacter was abundant in the K88 group. However, in the NA group, *Bacteroidetes*, and *Bacteroidia* were abundant. Enterobacteriales, proteobacteria and clostridium were relatively abundant in the K88 + NA group.

The relative abundance of top 10 in phylum, order and class were provided. Meanwhile, as shown in LEfSe, *Bacteroidetes*, *Bacteroidia*, and *Bacteroidales* were abundant among the four groups (Figures 5A–C). Results indicated that the relative abundance of *Bacteroidetes*, *Bacteroidales*, and *Bacteroidia* in the K88 + NA group was significantly reduced compared with the K88 group (*P* < 0.05).

### Effects of Nicotinic Acid on the Metabolomics in Colonic Contents of Weaned Piglets Infected by Enterotoxigenic *Escherichia coli*

**FIGURE 6:**
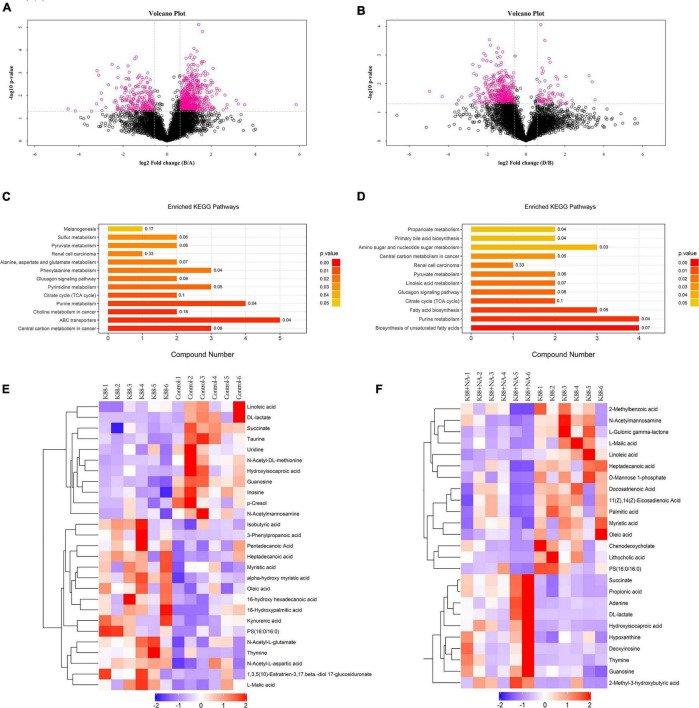
Metabolomic analysis of the colonic contents in weaned pigs caused by *E. coli* infection. Volcano plot in HILIC negative between K88 and Control **(A)** and negative between K88 + NA and K88 **(B)**; Enriched KEGG pathways analysis between K88 and Control **(C)**, between K88 + NA and K88 **(D)**; Hierarchical clustering of the differentiated metabolites in HILIC negative between K88 and Control **(E)**, between K88 + NA and K88 **(F)**.

Metabolomics analysis combined with enrichment analysis of colonic contents were shown in Figure 6 and Table 3. These results revealed that the K88 group altered the concentrations of metabolites (e.g., Isobutyric acid, Oleic acid, Succinate, Heptadecanoic acid, Cholic acid and so on) compared with Control group, and these metabolites were involved in ABC transporters and citrate cycle. In addition, the K88 + NA group altered the concentrations of metabolites (e.g., Propionic acid, Succinate, Hydroxy-isocaproic acid, Heptadecanoic acid, 2-Methyl-3-hydroxybutyric acid and so on) compared with the K88 group, and these metabolites were involved in ABC transporters and citrate cycle.

**TABLE 3:** Identified differential metabolites of the colonic contents in weaned pigs caused by *E. coli* infection.

Metabolite name	Positive/negative	VIP	Fold change	*p*-value
**K88 vs. Control**				
Guanosine	(M-H)-	1.63	0.38	0.0002
Inosine	(M-H)-	2.72	0.59	0.002
Succinate	(M-H)-	2.21	0.74	0.0064
Taurine	(M-H)-	1.17	0.31	0.0112
N-Acetyl-DL-methionine	(M-H)-	1.3	0.42	0.0123
Thymine	(M-H)-	1.3	2.49	0.015
PS(16:0/16:0)	(M-H)-	2.12	3.43	0.0233
Isobutyric acid	(M-H)-	3.95	1.51	0.0265
Oleic acid	(M-H)-	2.45	1.77	0.0278
DL-lactate	(M-H)-	1.08	0.21	0.0283
N-Acetyl-L-glutamate	(M-H)-	2.63	1.49	0.0352
Heptadecanoic acid	(M-H)-	3.65	2.01	0.0365
N-Acetylmannosamine	(M-H)-	1.44	0.53	0.0459
N-Acetyl-L-aspartic acid	(M-H)-	2.33	1.43	0.0492
Gly-His	(M + H-H_2_O) +	1.46	2.91	0.0027
1-Oleoyl-sn-glycero-3-phosphocholine	(M + Na) +	1.59	0.40	0.0036
Bilirubin	(M + H) +	3.18	3.09	0.0043
Taurine	(M + H) +	1.60	0.33	0.0062
Inosine	(M + H) +	1.83	0.60	0.0081
D-Proline	(M + H) +	4.99	0.37	0.0112
His-Pro	(M + H-H_2_O) +	1.64	2.32	0.0140
Cholic acid	(M + H-H_2_O) +	4.98	5.26	0.0267
Swainsonine	(2M + H) +	2.22	1.95	0.0497
**K88 + NA vs. K88**				
Propionic acid	(M-H)-	1.57	1.78	0.0029
Succinate	(M-H)-	4.91	1.85	0.0037
Hydroxyisocaproic acid	(M-H)-	4.44	10.62	0.0086
D-Mannose 1-phosphate	(M-H)-	1.00	0.30	0.0096
Oleic acid	(M-H)-	4.63	0.26	0.0196
N-Acetylmannosamine	(M-H)-	1.06	0.38	0.0335
Thymine	(M-H)-	3.08	5.17	0.0376
Guanosine	(M-H)-	1.55	1.86	0.0388
Hypoxanthine	(M-H)-	5.32	1.60	0.0398
2-Methyl-3-hydroxybutyric acid	(M-H)-	1.26	3.15	0.0438
Lithocholic acid	(M-H)-	7.51	0.20	0.0470
Pregnenolone sulfate	(M + H) +	1.00	0.44	0.0186

### Effects of Nicotinic Acid on the Expression of Intestinal Antibacterial Peptides in Weaned Piglets

**FIGURE 7:**
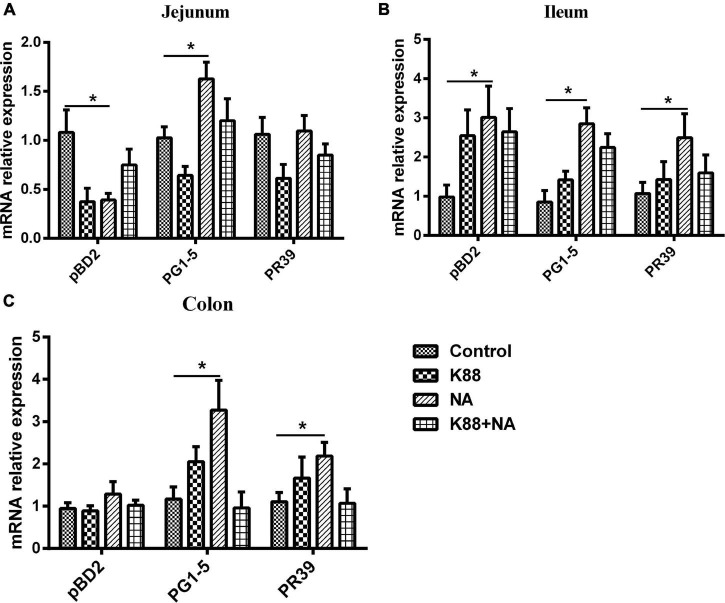
Nicotinic acid improved the expression of intestinal antibacterial peptides caused by *E. coli* infection of weaned piglets. Real-time quantitative PCR was performed to determine the relative mRNA expression levels of pBD2, PR39, and PG1-5 in the jejunum **(A)**, ileum **(B)**, and colon **(C)** of weaned piglets were detected by real-time PCR. All data are expressed as the mean ± SEM, *P < 0.05.

Further research is required to explore the effect of niacin on the expression of antimicrobial peptides in intestinal mucosa (Figures 7A–C). The NA group significantly improved the expression of antimicrobial peptide PG1-5 in jejunum, pBD2, PG1-5, and PR39 in ileum of weaned piglets compared with the Control group (*P* < 0.05). In addition, the NA group also significantly improved the expression of antimicrobial peptide PG1-5 and PR39 in colon of weaned piglets (*P* < 0.05).

### Effects of Nicotinic Acid on the Histone Acetylation Modification in Intestinal Mucosa in Weaned Piglets

**FIGURE 8:**
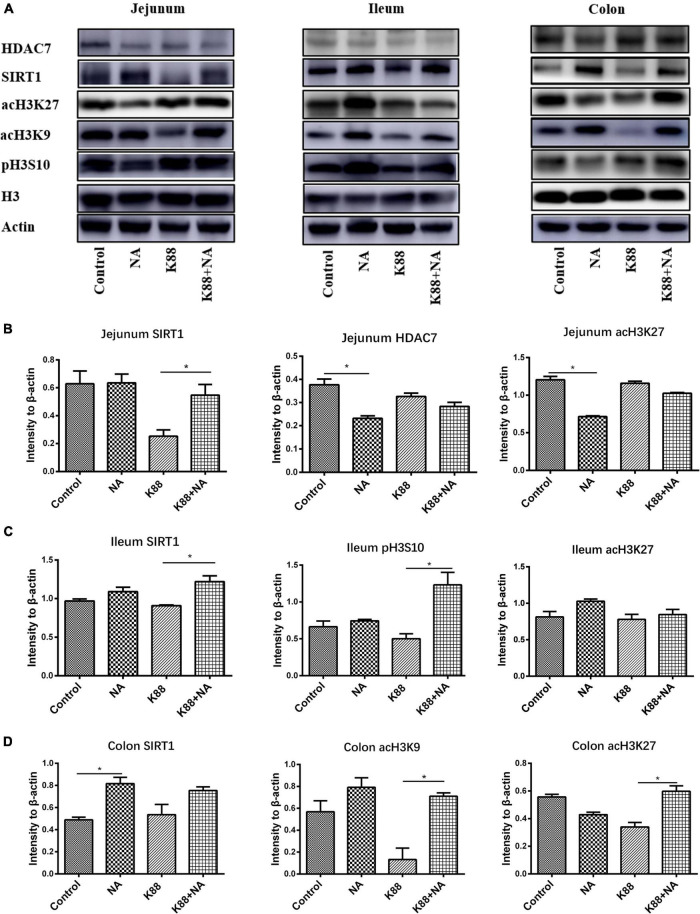
Effects of nicotinic acid on the histone deacetylase modification in intestinal mucosa caused by *E. coli* infection of weaned piglets. The expression of histone deacetylase SIRT1, HDAC7, and modification sites such as acH3K9, acH3K27, and pH3S10 in the promoter region in the jejunum, ileum, and colon mucosa were determined by Western blot **(A)**. The intensity of the bands was detected using ImageJ, densitometric values were normalized to those of β-actin in the jejunum **(B)**, ileum **(C)**, and colon **(D)**. *P < 0.05.

The effect of nicotinic acid on intestinal histone acetylation modification of ETEC infected piglets was further studied by Western blot (Figures 8A–D). The results showed that the NA group significantly reduced the level of HDAC7 in jejunum (*P* < 0.05) compared with the Control group. In addition, the K88 + NA group significantly increased the level of SIRT1 in jejunum (*P* < 0.05) compared with the Control group; Compared with the K88 group, the levels of histone SIRT1 and pH3S10 in ileum were significantly increased in the K88 + NA group (*P* < 0.05); The NA group significantly increased the level of SIRT1 in the colon (*P* < 0.05) compared with the Control group. Moreover, compared with the K88 group, the K88 + NA group significantly increased the levels of histone acH3K9 and acH3K27 in colon (*P* < 0.05).

## Discussion

Colibacillosis of weaned piglets caused by pathogenic *E. coli*, with severe diarrhea as the main clinical symptom, is one of the most serious diseases in China’s livestock industry (13). At present, ETEC is the most popular pathogenic bacteria in actual production of pigs. It colonized and proliferated in intestinal epithelial cells of weaned piglets through adhesion factors, thus destroying the normal balance of intestinal flora and causing secondary infections. Meanwhile, it could also release some enterotoxins to induce intestinal inflammation. These factors eventually resulted in diarrhea because of the unbalance of intestinal water and electrolyte metabolism (14). Whether the morphological structure of intestinal epithelium is intact or will not affect the normal intestinal mucosal immune response and barrier function (15). Villus height and crypt depth is an important index of the intestinal health of piglet. Previous studies found that villus height and crypt depth of duodenum and jejunum of weaned piglets were significantly increased after caused by *E. coli* infection (16). The present study focused on studying the effects of nicotinic acid on intestinal morphology and clinical symptoms caused by *E. coli* infection. We demonstrated that administration of nicotinic acid effectively improved mental state, attenuated intestinal tissue injury and weight loss. Moreover, the number of bacterial translocations in the liver and spleen of piglets was significantly reduced. Research has shown that nicotinic acid supplements in the diet not only improve the performance of weaned piglets, but also reduce the occurrence of diarrhea piglets (17). It was found that supplementation with tryptophan could alleviate the reduction in average daily gain (ADG) of piglets caused by *E. coli* infection (18). In addition, nicotinic acid supplements in the diet could increase the villus height of the small intestine in piglets caused by *E. coli* infection (19). In conclusion, nicotinic acid can effectively reduce the intestinal injury of ETEC K88 and prevent the infection of ETEC K88.

Due to the immature development of the intestinal immune system of weaned piglets, the intestinal mucosa of the weaned piglets is often susceptible to the invasion of some pathogenic bacteria, which causes intestinal inflammation (20). The main characteristic of intestinal inflammation is that inflammatory mediators are enriched in the intestinal mucosa. Cytokines play an important role in mediating intestinal tissue damage and coordinating inflammatory response, and are considered as the cornerstone of the body’s intracellular monitoring system. Cytokines are small molecule peptides or glycoproteins secreted by antigen-presenting cells (APCs) and have biological functions such as participating in inflammatory response and regulating immune response. Interleukin-6 (IL-6), tumor necrosis factor-α (TNF-α), and interferon-β (IFN-β) are important pro-inflammatory cytokines. These cytokines may be upregulated in response to inflammation in the body (21). Studies have confirmed that the mRNA expression of TNF-α, IL-6, IL-8, and other cytokine genes was increased in intestinal tissues of weaned piglets caused by *E. coli* infection (22, 23). The results of the current study showed that nicotinic acid supplements in the diet could ameliorate inflammation by down-regulated the expression of TNF-α, IL-6, and IL-8 in piglets caused by *E. coli* infection. This finding was partly consistent with the study by Kwon et al., who demonstrated that niacin alleviated pulmonary inflammation by reducing the expression of IL-6 and TNF-α in serum (24). Another study showed that niacin reduced the release of TNF-α, IL-6, and IL-8 by inhibiting the nuclear factor kappa-β (NF-κβ) signaling pathway in mouse alveolar inflammatory cells induced by lipopolysaccharide (LPS) (25). In addition, it is reported that niacin can alleviate intestinal inflammation by inhibiting the expression of inflammatory cytokines in mouse macrophages or reducing intestinal epithelial cell apoptosis (26). These data suggest that the defense of nicotinic acid against intestinal inflammation caused by ETEC K88 is mainly achieved by reducing TNF-α, IL6, and IL8.

Immunoglobulin is produced when the body is stimulated by antigens such as bacteria and viruses. It could make pathogens lose their pathogenic effect by reacting with antigens and blocking the harm of pathogens to the body (27). Secretory immunoglobulin A (SIgA), as mucosal humoral immune antibody, plays an important role in the local inflammatory response in the aspect of resisting external pathogenic microorganisms on mucous membrane of the host (28). Research has shown that pathogens will be easier to adhere and invade to the intestinal mucosa epithelium, causing enteritis and enterogenous systemic infection when the level of intestinal mucosa secretion SIgA is reduced (29). Our results demonstrated that nicotinic acid could significantly reduce SIgA with ETEC K88 infection. It suggest that nicotinic acid may resist ETEC K88 mainly by reducing the colonization of ETEC K88 in the intestine.

Intestinal microflora plays an important role in preventing pathogen adhesion and colonization (30), promoting digestion and metabolism, enhancing autoimmunity and maintaining health (31). If the intestinal microflora is out of balance, it may destroy the normal physiological function of the body, which will greatly increase the incidence of diseases, and thus lead to intestinal stress syndrome, inflammatory enteritis and diarrhea (32). *Bacteroidetes* and Firmutes are the dominant flora in the intestinal tract of piglets, accounting for more than 90% (33). With the change of growth environment, the dominant flora in the intestinal tract will change accordingly. Earlier report has shown that the increase of intestinal microbial diversity may enhance the stability of intestinal microflora, and thus enhance its ability to resist the invasion of pathogenic bacteria (34). It is shown that ETEC infection not only cause the imbalance of intestinal micro-ecological environment, but also significantly reduce the diversity of intestinal microflora (35). It is reported that the relative abundance of bacteroides is closely relative to the diarrhea rate of weaned piglets (36). In this study, the results show that nicotinic acid could significantly increase the diversity of microbial species in colon contents of weaned piglets. Meanwhile, compared with the K88 group, the K88 + NA group was significantly reduced the relative abundance of *Bacteroidetes* in phylum, class and order levels. There is accumulating evidence indicating that the metabolite of the intestinal microflora-butyrate could upregulate endogenous host defense peptides to enhance disease resistance in piglets (37, 38).

Antimicrobial peptides that were secreted by intestinal epithelial and immune cells of piglets not only play an important role in killing bacteria directly, regulating the immune system and immune regulation, but also improving the body’s resistance to pathogen infection by enhancing intestinal epithelial barrier function (39, 40). It has always been the focus of researchers that nutrition regulates the expression of intestinal antimicrobial peptides to improve resistance to disease. As far as we know, NA could alleviate intestinal inflammation and promote the expression of endogenous antimicrobial peptides. Some studies reported that NA could improve their ability of resistance to pathogen infection by improving the mice expression of antimicrobial peptide (CAMP and LF) (7). In addition, there is research has revealed that NA supplements in the diet could significantly improve the expression of antimicrobial peptides in the intestinal epithelial cells of mice, which could alleviate intestinal inflammation and diarrhea caused by the lack of tryptophan (41). In this study, we evaluated the effect of NA on the expression of antimicrobial peptides in intestinal mucosa. The results found that NA could significantly improve the expression of antimicrobial peptide PG1-5 in jejunum, pBD2, PG1-5, and PR39 in ileum, PG1-5 and PR39 in colon of piglets. NA increases the expression of AMPs might be one of the reasons for its defense against ETEC K88 infection. However, the regulatory mechanism that nicotinic acid improved the expression of intestinal endogenous antimicrobial peptides in weaned piglets is unclear, and further research is still needed to do.

Studies showed that histone modification could accurately regulate the expression of the innate immune response and the corresponding defense genes (42). Histone acetylation is of great importance for the transcriptional regulation of intestinal epithelial antimicrobial peptides (43). Already there is evidence that butyric acid could upregulated endogenous host defense peptides by inhibiting histone deacetylase to strengthen disease resistance of piglets (37). It has also been shown that some HDAC inhibitors can regulate the expression of antimicrobial peptide LL-37 in gastrointestinal cells by inhibiting histone deacetylase (44). Other study has shown that when HDAC7 is overexpressed in mice, it causes inflammation in macrophages in mice (45). SIRT-1 is an NAD-dependent deacetylase, and the changes of NAD counts *in vivo* will affect the activity of SIRT1 (46). Studies have found that nicotinamide can activate the NAD-sirtuins pathway to improve the expression of SIRT1 gene in the liver (47), and in some cases, NAM could act as an activator of SIRT1 (48). Another study found that SIRT1 activator (resveratrol) increased the expression of cathelicidin antimicrobial peptide (CRAMP) in mouse cells (49). The above research shows a correlation among nicotinic acid, antimicrobial peptides and histone acetylation modification. This suggests that nicotinic acid may regulate SIRTs and up regulate endogenous host defense peptides as a NAD supplement. In our study, SIRT1 mainly activated in jejunum and ileum during infection. But activated in colon without infection. This may be related to the different bacterial community. *ETEC K88* infection mainly caused the changes of bacterial community in the contents of foregut segment, so NA may be able to activate SIRT1 in jejunum and ileum. Furthermore, transcription factors enter chromatin to bind promoters of innate immune genes, requiring acetylation of histone H3 lysine residues and phosphorylation of histone H3S10 (50). In addition, some studies showed that HDAC inhibitors (TSA or SAHA) could significantly improve the expression of antimicrobial peptides (HBD2 and LL-37) in intestinal epithelial cells *via* increasing the acetylation level of histone H3K9 lysine residues (43). It was found that HDAC inhibitor (butyric acid) increased the expression of histone H3K9 in the antimicrobial peptide promoter region, thereby promoting the expression of antimicrobial peptides (PBD2 and PR39) in porcine macrophages (37). These results suggest that the elevated level of histone H3K9 in the promoter region may be a typical marker of transcriptional activation of antimicrobial peptide genes. In our study, we found that nicotinic acid associated with the histone deacetylase SIRT1 and HDAC7, improving the histone modification sites H3K9, acH3K27 and pH3S10 in the promoter region. Taken together, these results suggest that nicotinic acid may increase the expression levels of acH3K9, acH3K27, and pH3S10 in the promoter region by activating intestinal histone deacetylase SIRT1 or inhibiting HDAC7, and then further up-regulated the expression of endogenous AMPs in weaned piglets.

## Conclusion

In conclusion, NA could alleviate the clinical symptoms, the damage of intestinal morphology, and intestinal inflammation in weaned piglets infected ETEC K88, and NA may improve intestinal antimicrobial peptides to enhance resistance of *E. coli* infection by regulating intestinal microflora and its metabolites, histone deacetylase SIRT1 and HDAC7, histone modification sites (acH3K9, acH3K27, and pH3S10) in the promoter region.

## Data Availability Statement

The datasets presented in this study can be found in online repositories. The names of the repository/repositories and accession number(s) can be found at: https://www.ncbi.nlm.nih.gov/, SRP357346.

## Ethics Statement

The animal study was reviewed and approved by the Animal Care Committee of the Institute of Animal Science, Guangdong Academy of Agricultural Sciences.

## Author Contributions

RZ, JF, LW, and HY designed the study and contributed to the revision of the manuscript. JF, DH, TC, WC, YC, YX, YQ, and ZJ performed the research. JF analyzed the data and wrote the manuscript. All authors read and approved the final manuscript.

## Conflict of Interest

The authors declare that the research was conducted in the absence of any commercial or financial relationships that could be construed as a potential conflict of interest.

## Publisher’s Note

All claims expressed in this article are solely those of the authors and do not necessarily represent those of their affiliated organizations, or those of the publisher, the editors and the reviewers. Any product that may be evaluated in this article, or claim that may be made by its manufacturer, is not guaranteed or endorsed by the publisher.
